# Abnormal Expression of Mitochondrial Ribosomal Proteins and Their Encoding Genes with Cell Apoptosis and Diseases

**DOI:** 10.3390/ijms21228879

**Published:** 2020-11-23

**Authors:** Guomin Huang, Hongyan Li, Hong Zhang

**Affiliations:** 1 Department of Medical Physics, Institute of Modern Physics, Chinese Academy of Sciences, Lanzhou 730000, China; huangguomin@impcas.ac.cn (G.H.); lihy@impcas.ac.cn (H.L.); 2 Advanced Energy Science and Technology Guangdong Laboratory, Huizhou 516029, China; 3 Key Laboratory of Heavy Ion Radiation Biology and Medicine of Chinese Academy of Sciences, Lanzhou 730000, China; 4 College of Life Sciences, University of Chinese Academy of Sciences, Beijing 101408, China; 5 School of Nuclear Science and Technology, University of Chinese Academy of Sciences, Beijing 101408, China

**Keywords:** mitochondrial ribosome, mitochondrial ribosomal proteins, apoptosis, cancer, mitochondrial disease, biomarker

## Abstract

Mammalian mitochondrial ribosomes translate 13 proteins encoded by mitochondrial genes, all of which play roles in the mitochondrial respiratory chain. After a long period of reconstruction, mitochondrial ribosomes are the most protein-rich ribosomes. Mitochondrial ribosomal proteins (MRPs) are encoded by nuclear genes, synthesized in the cytoplasm and then, transported to the mitochondria to be assembled into mitochondrial ribosomes. MRPs not only play a role in mitochondrial oxidative phosphorylation (OXPHOS). Moreover, they participate in the regulation of cell state as apoptosis inducing factors. Abnormal expressions of MRPs will lead to mitochondrial metabolism disorder, cell dysfunction, etc. Many researches have demonstrated the abnormal expression of MRPs in various tumors. This paper reviews the basic structure of mitochondrial ribosome, focuses on the structure and function of MRPs, and their relationships with cell apoptosis and diseases. It provides a reference for the study of the function of MRPs and the disease diagnosis and treatment.

## 1. Introduction

Ribosome is the ribonucleoprotein particle, which is an organelle for protein synthesis in cells, and its function is to synthesize the polypeptide chain efficiently and accurately according to the information of mRNA. Ribosomes can be found in almost all cells and even the smallest and simplest mycoplasma cell contains hundreds of them. At present, only mammalian mature red blood cells have no ribosomes, therefore, ribosomes are an indispensable structure of most cells. Mitochondria and chloroplasts contain ribosomes that synthesize their own proteins, which may be related to the origin of mitochondria and chloroplasts. The accepted endosymbiotic origin theory holds that mitochondria and chloroplasts originated from symbiotic bacteria and cyanobacteria in primitive eukaryotic, respectively. Therefore, mitochondrial ribosomes are more similar to the bacterial ribosomes than to cytoplasmic ribosomes. Mitochondrial ribosomal proteins (MRPs) are encoded by nuclear genes and synthesized by the cytoplasm 80S ribosomes, after specific targeting, sorting, transporting to mitochondria, and then assembling into mitochondrial ribosome small and large subunits with two rRNAs encoded by mitochondrial DNA (mt-DNA). With the research on MRPs, their names have changed [1], as shown in Table 1. The table also shows the changes of MRPs in tumor tissues reported in the past 5 years.

In this paper, we will introduce the basic structure of mitochondrial ribosomes and emphasize the significance of MRPs in mitochondria. Some of the MRPs can participate in cell apoptosis as apoptosis-inducing factors or play a functional role in the process of tumorigenesis. We will highlight the relationship between MRPs and diseases, and the important influence of MRPs as molecular markers in relevant fields.

## 2. MRPs on Structure and Function of Mitochondrial Ribosomes

**Figure 1:**
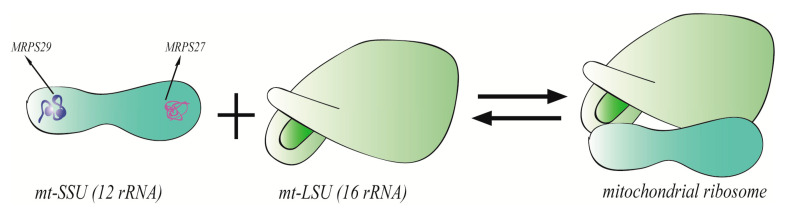
Mitochondrial ribosomes are composed of mt-SSU and mt-LSU. The overall structure of mt-SSU is divided into three areas: head, platform, and foot. The head region is characterized by the presence of MRPS29 and the foot region is characterized by MRPS27.

The mitochondrial ribosome is an irregular granular structure without biofilm encapsulation, its main components are RNA (account for two-thirds) and proteins (account for one-third). Protein is mainly distributed on the surface of ribosomes and RNA is mainly located in the interior, they are held together by non-covalent bonds. The mitochondrial ribosome is 25–30 nanometers in size and contains two different subunits, mt-LSU and mt-SSU. The mt-SSU is made of 12S rRNA and 30 MRPs, the mt-LSU consists of 16S rRNA, mt-tRNA^Val^, and 50 MRPs. The 12S rRNA and 16S rRNA are transcribed from the mt-DNA gene and 36 of the 80 proteins are specific to the mitochondria. The overall structure of mt-SSU is elongated and divided into three areas: head, platform, and foot (Figure 1). The head region is characterized by the presence of MRPS29 and the foot region is characterized by the pentapeptide repetition (PPR) domain protein of MRPS27 [79]. The mt-LSU contains mt-RNA^Val^, which interacts with several MRPs such as MRPL38 and MRPL40 at the central protuberance (CP). The CP is a functional landmark of the large subunit, which mediates inter-subunit contacts to mt-SSU [80]. Moreover, mitochondrial ribosomes are connected to the mitochondrial inner membrane through large subunits.

The mitochondrial ribosome is highly similar to other known mitochondria and even replaces their homologues in bacteria functionally. However, during the long evolution, mitochondrial ribosomes undergo a series of reconstructions, they lack some of the main RNA structures compared to bacterial ribosomes. Overall, mitochondrial ribosomes contain about half as much rRNA as bacterial ribosomes but almost twice as much as protein [81,82]. The increase in protein mass results in longer subunits and a wider protein network of more than 200 contacts. Some studies claimed that special proteins in the mitochondrial ribosomes fill in the missing RNA [83,84,85]. However, different results have emerged in recent years, in which a high-resolution cryo-EM reconstruction of mammalian mitochondrial ribosomes revealed that the MRPs extension predominantly interacts with mitochondrion-specific proteins, whereas only a few participate in filling the space of the deleted rRNA [79,86].

**Figure 2:**
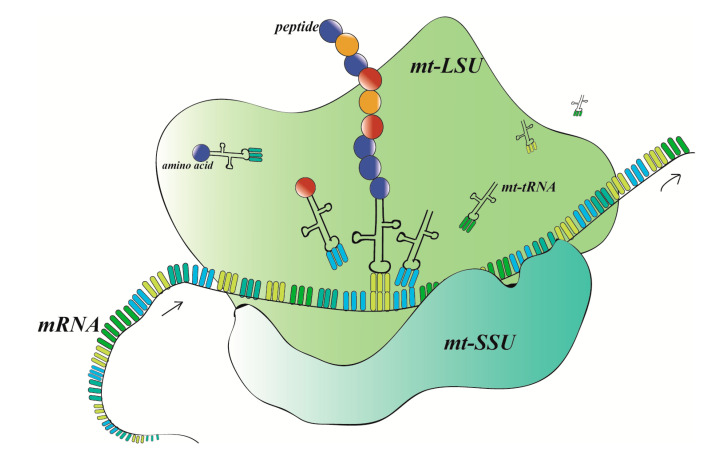
The function of mitochondrial ribosome is to accurately synthesize the polypeptide chain from the mRNA information of mitochondrial DNA transcription.

Mitochondrial ribosomes produce proteins correctly and efficiently, and their biosynthesis and transcriptional control are essential cellular processes that can be regulated at multiple levels. The mt-SSU provides a platform for mRNA binding and decoding, while mt-LSU catalyzes the peptide-transferase reaction. The A-site residues contributed by the mt-SSU are important for decoding and the mt-LSU has maintained the ability to mediate the main contact between mRNA and 16S rRNA [85] (Figure 2). The protein function of mitochondrial ribosomes is conserved. However, the mammalian mitochondrial ribosomes have substantial differences in both the entry and exit sites of this channel compared to other ribosomes. At the entry site, they lack protein MRPL4 and a domain of MRPL3 compared with bacteria. This missing part may be replaced by MRPL5, which connects the mt-SSU and head domains to form a latch across the mRNA channel [85]. The mRNAs enter the channel entrance as a single strand and bind to PTCD3 (MRPS39), the largest protein of the mt-SSU. In addition, MRPS39 knockdown results in a significant overall decrease in mitochondrial protein synthesis [87]. During translation initiation, the 3′ end of rRNA is stably associated with MRPS37 [88]. Another obvious difference is the polypeptide exit tunnel. Generally, the exit region of the tunnel is defined by a conserved ring of proteins (MRPL22, MRPL23, MRPL24). However, in mammals, a second layer of additional mitochondrial specific proteins (MRPL39, MRPL44, MRPL45) are found extending the conserved ring. Only mitochondrial ribosomes acquire an intrinsic GTPase activity through MRPS29, which is the signature protein of the mt-SSU head and its deficiency results in shrunken mitochondria [89]. The foot signature protein (MRPS27) is associated with 12S rRNA and tRNA. Additionally, this protein is necessary for mitochondrial translation since knocking it out reduces the abundance of respiratory complexes and the activity of cytochrome C oxidase [90].

## 3. The Relationship between MRPs and Cell Apoptosis

Mitochondria play crucial roles in the induction of apoptosis or programmed cell death. In addition, an altered mitochondrial function and defective apoptosis are well-known hallmarks of cancer cells. Three MRPs associated with apoptosis have been identified, namely, MRPS29 (DAP3), MRPL41 (BMRP), and MRPS30 (mL65).

### 3.1. MRPS29 (DAP3)

MRPS29, also known as death-associated protein 3 (DAP3), is a GTP-binding protein and a head marker of mt-SSU [91]. MRPS29 contains three potential structural motifs for GTP binding, including the phosphate binding P-loop motif, which is essential for MRPS29-induced cell death [92]. Therefore, MRPS29 is involved in the alterations of the mitochondrial network dynamics that occur during apoptosis, cell death, and mitochondrial rupture [89,93]. A high expression of MRPS29 promotes apoptosis [94]. MRPS29 has been found on the outside of mitochondria to initiate the extrinsic apoptotic pathway through its interactions with apoptotic factors such as Fas ligand, tumor necrosis factor-alpha (TNF-α) and interferon-gamma (IFN-γ) [95]. In this process, MRPS29 does not have to be released from the mitochondria to perform its cell death function [96]. DAP3 binds directly to the death domain of TNF-related apoptosis-inducing ligand (TRAIL) receptors. Moreover, DAP3 associates with the pro-caspase-8–binding adapter protein Fas-associated death domain (FADD), and links FADD to the TRAIL receptors, DR4 and DR5 [97,98]. Furthermore, MRPS29 is a substrate of the kinase AKT (PKB), while AKT opposes the pro-apoptotic action of MRPS29 [99].

### 3.2. MRPL41 (BMRP)

MRPL41 has also been named BMRP (Bcl-2 interacting mitochondrial ribosomal protein) [100]. In the structure of the mt-LSU, MRPL41 has most of the extended conformation and interacts with 16S rRNA and several surrounding MRPs, including another pro-apoptotic protein, MRPL65 [84].

MRPL41 could perform the function of binding and participating in the regulation of the PTP/Bcl-2 mitochondrial complex. It is released from the mitochondria and interacts with both the BH4 domain and the central region encompassing BH1, BH2, and BH3 domains of Bcl-2, rather than initiating apoptosis in the mitochondria [100,101]. The cell death induced by MRPL41 is also inhibited by the caspase inhibitor p35, suggesting that BMRP induces apoptosis by a mechanism mediated by caspases, which can be blocked by Bcl-2 or the baculoviral caspase inhibitor p35 [100]. Additionally, overexpression of MRPL41 enhances the p53 stability and makes it translocated to the mitochondria, therefore, contributing to the p53-induced apoptosis (via transcription independent mechanisms) in response to growth-inhibitory conditions [102]. In the absence of p53, MRPL41 has also been shown to mediate a serum starvation-induced cell cycle arrest through an increase of p21^WAF/CIP1^ and p27^Kip1^ protein levels, revealing that MRPL41 can induce apoptosis through the p53-independent pathway [103].

### 3.3. MRPL65 (PDCD9, MRPS30)

MRPL65 was also known as PDCD9 (programmed cell death protein 9) or MRPS30, it was originally considered to be a small subunit protein. But it was later observed to appear on the large subunit, and therefore, renamed mL65 [92]. Overexpression of mrpl65 in mouse fibroblasts can induce apoptosis by upregulating transcription factors c-Jun and activating the c-Jun N-terminal kinase 1 (JNK1) [104]. However, little is known overall about the molecular mechanisms underlying the pro-apoptotic roles of mL65 up to now.

## 4. MRPs Associated with Cancers

Scientists explored the new mutations, epigenetic disorders, abnormal genes expression and protein abundance patterns based on genomics and proteomics, which establish causal relationships between MRPs abnormalities and carcinogenesis or provide diagnostic and therapeutic markers in some cases [2,105]. Based on additional experimental results, the same MRPs may affect multiple cancers and multiple MRPs abnormalities can be detected in the same cancer, which forms a complex and changeable network system. MRPs may be used as markers for the detection of the development of various cancers. However, the specific mechanism of MRPs-induced cancer is still lacking, and the way in which they play a role in cancer needs to be further explored.

### 4.1. MRPs and Breast Cancer

The most widespread concern is the relationship between MRPs and breast cancer. Genome-wide association studies (GWASs) have revealed an increased breast cancer risk, which is associated with multiple genetic variants in 5p12 [106]. The risk allele, rs4415084-T, is highly correlated with the MRPS30 expression level through a new method combining a quantitative expression trait locus analysis and allele-specific expression analysis in the 5p12 breast cancer susceptibility region [107]. Based on another evidence, the 5p12 variant, rs10941679, was regulated by *FGF10* and *MRPS30* to increase susceptibility to estrogen receptor positive breast cancer [106]. These ideas have been tested to a certain extent, using luciferase reporter assays in both estrogen-receptor positive (ER+) and negative (ER-) cell lines. Guo et al. showed that alternative alleles of potential functional single-nucleotide polymorphisms (SNPs), rs3747479 (*MRPS30*), could significantly change promoter activities of their target genes compared to reference alleles. MRPS30 plays a crucial role in the development of breast tumors in vitro [108]. *MRPS30-DT* (the long non-coding RNA, lncRNA) knockout significantly inhibits the proliferation and invasion of breast cancer cells, and induces cells apoptosis [109]. In addition to the 5p12 site, a new breast cancer susceptibility site was identified in 4q21 (rs11099601). An expression quantitative trait locus (QTL) analysis in breast cancer tissue showed that rs11099601 is associated with *MRPS18-C* [70].

In the process of exploring the relationship between mitochondrial biogenesis related nuclear coding genes and breast cancer recurrence, distant metastasis and prognosis, twelve different components of large subunit proteins showed a significant prognostic value, among which MRPL15 had the best prognostic value [16]. Similarly, the bioinformatics analysis found that MRPL13 may have a prognostic value for breast cancer, the survival rate of breast cancer patients with a high expression of MRPL13 was relatively low, which could be used as a potential prognostic marker for breast cancer [13,14,15]. MRPL33, a protein in mt-LSU, is linked with breast cancer metastasis through RNA depth sequencing (RNA-seq) analysis and the content of MRPL33 exon 3 was significantly increased in breast cancer, lung cancer, colon cancer, etc. [28]. The isoform-specific knockdown of exon 3-containing *MRPL33* mRNA (*MRPL33-L*) inhibits cancer cell growth and considerably induces cell death. The mechanism analysis showed that *MRPL33* exon 3 could be regulated by multiple splicing factors including the hnRNPK protein. The hnRNPK and *MRPL33-L* are required for a normal mitochondria function in cancer cells, as both depletion of them led to reduced levels of 16S rRNA and caused excessive reactive oxygen species (ROS) production and insufficient ATP generation [29]. In a recent study on the prediction and classification of breast cancer proteins, MRPL54 is one of the RNA-binding proteins associated with breast cancer [8]. A new logistic regression model to classify breast cancer tumor samples based on microarray expression data was introduced in some papers, which illustrated that those genes, such as *MRPL9*, exhibit oncogenic characteristics and may be potential breast cancer predictors [8]. Moreover, Zhang et al. also found that MRPL12 expression is upregulated in cancerous stromal cells compared with the cells in a normal breast tissue [11].

Several MRPs are associated with breast cancer, which are either differentially expressed specifically in breast cancer or interact with the breast cancer susceptibility gene loci to promote or inhibit the development of breast cancer. However, no clear pathway shows how MRPs play role in breast cancer. Subsequent studies would further explore the specific mechanisms of MRPs affecting breast cancer.

### 4.2. MRPs and Digestive Tract Cancers

Cancers of the digestive tract mainly include malignant tumors of the throat, esophagus, liver, gallbladder, pancreas, large and small intestines. With abundant achievements in the past 5 years, the relationship between MRPs and digestive tract cancer has been gradually revealed.

LncRNA *MRPL23* antisense RNA1 (*MRPL23-AS1*) is implicated in different cancers by an increasing microvascular permeability and promoting the metastasis of salivary adenoid cystic carcinoma in vivo, which also plays a role in the regulation of oral squamous cell carcinoma [24,110]. *MRPL33* has also been shown to be significantly associated with the human papillomavirus associated oropharyngeal squamous cell carcinoma [32].

Liver cancer is the fourth most common cancer in China. Therefore, the relationship between the development of liver cancer and MRPs has received much attention and was relatively clarified. In hepatocellular carcinoma, the decreased expression of MRPL13 is a key factor in the regulation of mitochondrial ribosome and subsequent OXPHOS (oxidative phosphorylation) deficiency, which regulated the aggressive activity of liver cancer cells [13]. However, MRPS23 overexpression was found to promote hepatocellular carcinoma cell proliferation and suggested a low survival rate in hepatocellular carcinoma patients [77]. Similar to MRPS23, the increase of MRPS18-A can promote the development of liver cancer [60]. According to the Pearson correlation analysis of the construction module and clinical traits, gene MRPS18-A affects not only the liver function but also the overall survival of patients with cholangiocarcinoma [61]. The CR6 interaction factor 1 (CRIF1, MRPL64) inhibits invasiveness by inhibiting TGF-β mediated epithelial mesenchymal transition in hepatocellular carcinoma [54]. Exon sequencing was performed on liver cancer samples using next-generation sequencing technology, the results indirectly proved that *MRPL38* was highly expressed in liver cancer [38].

Gastric cancer is the third most common cancer in China, which harms many people’s lives and health. If a reliable link can be found between MRPs and gastric cancer, it will provide a powerful help for the treatment of gastric cancer. In the treatment of gastric cancer with epriubicin, the MRPL33-short isomer (MRPL33-S) and MRPL33-long isomer (MRPL33-L) showed opposite effects. MRPL33-S promoted the sensitivity of gastric cancer cells to epirubicin, while the splicing variant MRPL33-L inhibited this effect. The upregulated MRPL33-S could promote the chemotherapy response of gastric cancer cells to epirubicin, whereas MRPL33-L suppressed the chemotherapy response [30]. Sotgia et al. interrogated 5-year follow-up data collected from a group of N = 359 gastric cancer patients and combined MRPL28 and other eight mitochondrial proteins to generate a compact gastric mitochondrial gene signature [27]. Another study has represented that MRPL39 serves as a tumor suppressor by directly targeting miR-130 in gastric cancer, suggesting that it may be a new gastric cancer biomarker for diagnosis and prognosis [39]. The expression level of MRPL43 in gastric cancer tissues is significantly upregulated. However, no obvious difference is seen in colorectal carcinoma (CRC), lung cancer and papillary thyroid cancer [44].

The incidence of colorectal cancer in China is only next to lung cancer. CRIF1 (MRPL64) enhanced the p53 activity in HCT116 colon cancer cell lines through chromatin remodeling SNF5, thereby inhibiting cell growth and tumor development [111]. *MRPL12* as a downstream gene of c-Myc participates in the cetuximab resistance in RAS wild-type colorectal cancer (CACO2-CR) cells [12]. MRPS18-2 may be a biomarker for CRC based on the analysis of the genetic polymorphism of Gly132Cys in clinical CRC samples and surrounding normal tissues. However, the function of this polymorphism and potential of MRPS18-2 as a CRC biomarker still needs to be assessed [68]. In addition, mrpl35 is upregulated in CRC and regulates the growth and apoptosis of CRC cells, which may be a potential therapeutic target for CRC [35]. *MRPL52* is able to significantly predict the survival of patients with CRC, which is downregulated in the poor survival colorectal cancer sample [52].

In fact, there are numerous studies on the relationship between MRPs and digestive tract cancer, some of the explorations have been done in the last 5 years. As mentioned above, *MRPL23* not only promotes the metastasis of salivary adenoid cystic carcinoma, it is also related to the occurrence of liver cancer [112], suggesting that the same MRP can affect different cancers. However, it is not clear whether there is a difference in the mechanism of its influence. In the future, understanding the abnormal expression of different MRPs in the same cancer or a particular MRP in different cancers, would be of great help to the research of new treatment regimens.

### 4.3. MRPs and Other Cancers

Lung cancer development, with its high morbidity and mortality, is associated with an abnormal expression of MRPs. Maiuthed et al. used bioinformatic analysis and pharmacology experiments to verify the underlying regulatory mechanisms of MRPs on lung cancer and found a more than 5-fold increase in MRPL51 expression in a lung cancer cell model [51]. A new periodic mutation *MRPL1* (Tyr87Cys) was found in asbestos-induced lung cancer patients but not in normal samples [2]. Academics screened the differentially expressed mitochondrial ribosomal genes between iAs (inorganic arsenic)-treated lung cancer cells and controls. Based on microarray data analysis, they identified four ribosomal genes similar to *MRPL17* as key genes for prognosis of lung cancer [18]. A recent study found that MRPS16 promoted the growth, migration, and invasion of glioma cells by activating PI3K/AKT nail axis, these processes can be eliminated by knocking down *MRPS16* and similar trends are observable in U251 and A172 cells after deletion of *MRPL42* [43,113]. However, the high expression of MRPL35 can predict the longer survival of glioblastoma multiforme [34]. Benzyl isothiocyanate (BITC) inhibits several mitochondrial ribosomal genes including *MRPS28*, *MRPS2, MRPL23, MRPS12, MRPL12* and *MRPS34*, suggesting that these genes may be potential biomarkers for the treatment of glioblastoma by BITC [25].

In cancers of the blood system, MRPL33 affects the receptor tyrosine kinase TrkA or KIT expression levels in acute myeloid leukemia (AML) and neuroblastoma, which has a prognostic value for both types of cancer [31]. In the study of the first recurrence of AML patients, AML cells were significantly characterized by increased levels of mitochondrial functional important proteins such as MRPL21 and MRPS37, which may guide treatment strategies to reduce the chemotherapeutic resistance to recurrent AML [23]. Additionally, MRPL49 is also associated with AML prognosis [114]. MRPL47 variation is a risk factor for vincristine-induced peripheral neuropathy in children with acute lymphoblastic leukemia [50]. For diffuse non-Hodgkin lymphoma, *MRPL19* is one of the related genes and the geometric mean of its expression can be used to normalize gene expression in melanoma [21,22].

MRPs have a significant effect in female-specific cancers. *MRPL19* is one of the internal reference genes for type I or II endometrial cancers and the expression level of *MRPL46* had the greatest influence on the prognosis of ovarian cancer [19,20,49]. Overexpression of MRPS18-B (MRPS18-2) leads to the epithelial cells that acquire a higher migration ability to transform as mesenchymal cell (EMT). Further studies have shown that MRPS18-2 induces EMT through the TWIST2/E-cadherin signal and CXCR4 (PC3 cells express the chemokine receptor) mediates the migration of prostate cancer cells [67]. Lately, *MRPS7* was detected as a key gene in the pathophysiological process of osteosarcoma and the *MRPL3* content is positively correlated with the prognosis of osteosarcoma [6]. Through a bioinformatics analysis such as gene ontology, *MRPS11* was found to be overexpressed in uveal melanoma and is a potential prognostic indicator for uveal melanoma [65].

## 5. MRPs Associated with Diseases Except Cancers

MRPs genes are located at chromosomal sites and associated with a variety of human mitochondrial diseases. Mutations of MRPs genes can lead to diseases of varying degrees since the mitochondria cannot maintain a normal metabolic function. Changes in MRPs expression inhibits the mitochondrial genetic material translation (mainly mitochondrial respiratory chain protein synthesis) that destroys the mitochondrial ribosome composition, leading to mitochondrial diseases. In addition, MRPs can also act directly in some mitochondrial diseases through their own function changes. The defects in mt-DNA transcription and translation are associated with numerous classical mitochondrial diseases including mitochondrial encephalopathy, Leigh syndrome, mitochondrial neuro-gastro-intestinal encephalomyopathy, Pearson syndrome, etc. [46], which are closely related to the failure of mitochondria to maintain a normal metabolic function.

### 5.1. MRPs Associated with Mitochondrial Diseases

The deletion of MRPL10 can reduce the mitochondrial activity and expression of the mitochondrial complex, suggesting that MRPL10 is necessary for mitochondrial protein synthesis and mitochondrial activity. Moreover, the deletion of MRPL10 inhibits the kinase activity of cyclin-dependent kinase CDK1 and promotes mitochondrial fusion through dephosphorylation of Drp1^Ser616^, thereby affecting adaptive metabolic response, cell proliferation, and cell survival [115]. By analyzing the genomes of 142 patients with complex defects in the mitochondrial respiratory chain, *MRPS23* was confirmed to be one of the genes responsible for the defect of the mitochondrial respiratory chain complex [116]. In addition, human *MRPS34* mutations lead to Leigh or Leigh-like syndrome by destabilizing the mt-SSU and impairing mitochondrial protein translation [117]. Similarly, *MRPS5* mutation affects the accuracy of mitochondrial ribosome assembly, leading to stress-related behavioral changes. Moreover, there is evidence that the homozygous mutant *mrps5* V338Y mice was sensitive to noise since hearing loss and anxiety related behavior changes [62]. CRIF1 (MRPL64) is a very important component of the mt-LSU. The conditional knockout of *crif1 (mrpl64)* in specific tissues (such as brain, heart, intestine and adipose tissues) of mice induces mitochondrial dysfunction, resulting in epidermal damage skin homeostasis and hair morphogenesis [118]. The skeletal muscle deficiency of crif1 (mrpl64) induces progressive mitochondrial OXPHOS dysfunction and UPR^mt^ activation, eventually resulting in late muscular dystrophy and sarcopenia [119]. In the study, regarding whether and how the lymphocyte-specific protein tyrosine kinase (Lck) promotes the metabolic transfer of T-cell leukemia through mitochondrial localization, mitochondrial Lck interferes with the mitochondrial translation mechanism by competitive binding to CRIF1 (MRPL64) [55]. Furthermore, the crif1 (mrpl64) deficiency promotes endothelial inflammation by downregulating SIRT1 and inducing vascular inflammation [57,58]. In mice with an impaired mitochondrial oxidative function of macrophages, a bone marrow-specific loss of crif1 (mrpl64) gene function causes systemic insulin resistance and lipo-inflammation [120]. In addition to *crif1 (mrpl64),* the mutation of *mrpl20* was most pronounced in TALLYHO/Jng (TH) mice (the mice mimics many characteristics of human non-insulin-dependent type II diabetes mellitus) and may alter the folding, structure, and activity of the protein, as well as the ability of mrpl20 to interact with other MRPs in the mitochondrial ribosome [121]. These redundant residues lead to an impaired mitochondrial biogenesis and abnormal OXPHOS. Therefore, *mrpl20* is a key gene, which induces defective mitochondrial protein synthesis in TH mice [121]. In addition, mrps23, mrpl27, mrpl45 and mrpl48 mRNA levels are observed to be elevated in the heart and decreased in the liver of rats with metabolic syndrome [122]. The structural variation of *mrpl3* induces neurodegeneration and memory impairment in adult mice [3].

### 5.2. Clinical Manifestations of MRPs Related Mitochondrial Disease

MRPs are associated with a range of mitochondrial diseases, in which clinical manifestations are complex. A homozygous mutation of MRPS22 was detected in a prepubertal woman with blepharophimosis, ptosis and epicanthus inversus syndrome (BPEs), resulting in a soft palate cleft and microcephaly other than the BPEs phenotype [72]. Homozygous mutations in *MRPS7* were associated with primary hypogonadism, primary adrenal failure, sensorineural deafness, as well as lactic academia [64]. Likewise, biallelic mutations in *MRPS2* also caused sensorineural hearing loss, hypoglycemia and multiple OXPHOS complex defects [123]. In a Japanese patient with Leigh syndrome, the mutations of the biallelic *PTCD3 (MRPS39)* resulted in frameshift changes and generated premature stop codons. Moreover, the loss function of *PTCD3* resulted in translation defects in the mt-DNA encoding protein, accompanied with OXPHOS deficiency and destabilization of the mt-SSU. These findings suggested that *PTCD3* mutations are related to mitochondrial ribosomal protein defects, causing neurodegenerative disease and premature death [78].

A homozygous missense mutation of *MRPL24* was detected in one female infant with cerebellar atrophy, limb or facial arrangement and intellectual disability. In addition, complex I and IV defects were found in her muscle biopsy [26]. The regulation of *MRPS18-C* (encoding bS18m protein) expression might be an effective treatment strategy through studying fibroblasts from an epileptic encephalopathy patient with the m.3946G>A mutation in the mt-ND1 gene [71]. Significant reductions in *MRPL11* and *MRPL44* expression were found in the fibroblasts of another female infant with symptoms of mitochondrial encephalopathy, such as neonatal-onset seizures, microcephaly, pachygyria, progressive white matter atrophy and cortical blindness [10]. A few studies have reported that *MRPL40* haploinsufficiency induced short-term potentiation abnormalities through mitochondrial-mediated deregulation of presynaptic Ca^2+^ levels, but did not lead to long-term plasticity or long-term memory [42]. The *MRPS28* variants lead to intrauterine growth retardation, craniofacial dysmorphism and delayed development. In addition, the disease-causing variants in the *MRPS28* were clarified [124]. A comprehensive analysis of major databases and literature showed that *MRPS22* is one of the genes associated with the genetic etiology of primary ovarian insufficiency [74,75,76]. A few other key mitochondrial ribosomal genes have also been gradually elucidated. For example, *MRPS30* is one of the potential gene loci for sporadic Parkinson’s disease in Chinese mainland Han population [125]; *mrpl4* is one of the potential targets for the treatment of hypertension and stroke [126]; and *MRPS25* mutation results in dyskinetic cerebral palsy and partial agenesis of the corpus callosum [127].

Mitochondrial related diseases caused by MRPs were present at all ages. The clinical characterization of diseases caused by the same MRP is not single and often synergistic with other proteins, making it difficult to identify the specific clinical characterization of MRP diseases. The functions of MRPs in regulating various mitochondrial diseases would be revealed gradually based on a large amount of studies in the future.

### 5.3. MRPs and Heart Diseases

As the main site of intracellular OXPHOS and ATP formation, mitochondria are important organelles that control and regulate metabolism. Abundant mitochondria with normal MRPs expression play crucial roles in maintaining the heart’s function. An abnormal MRPs expression causes heart disease including hypertrophic cardiomyopathy and blood clots, since MRPs are necessary for the mitochondrial respiratory chain complex to function properly.

A variant in *MRPS14* caused perinatal hypertrophic cardiomyopathy with neonatal lactic acidosis, growth retardation, dysmorphia and neurological involvement. However, surprisingly, the mutant *MRPS14* was stable and did not affect the assembly of the mt-SSU [66]. *MRPL44* associated with cardiomyopathy has reported that *MRPL44* had mutation c.467T>G, (p.Leu156Arg) and c.233G>A, (p.Arg78Gln) in two unrelated patients with childhood hypertrophic cardiomyopathy. Similarly, the *MRPL44* mutation was described in two Finnish siblings aged 6 months and 14 years, respectively. The presented hypertrophic cardiomyopathy, as a leading symptom, was accompanied by the heart tissue with respiratory complex I and complex IV deficiency and fibroblast separation complex IV deficiency [128]. The *MRPL44* mutation was one of the important causes of cardiomyopathy and it resulted in mitochondrial cardiomyopathy in human infants, which is based on the analysis of 66 myocardial cases in the Finnish heart transplant center [45,46]. Moreover, the *MRPL44* mutation not only resulted in this trait, but also caused hemiplegia migraine, pigmentary retinopathy and renal insufficiency Leigh-like lesions on brain MRI (magnetic resonance imaging) [47].

The mutation of *MRPS22* is noticed to cause hypertrophic cardiomyopathy and fallopian tube lesions in a Turkish patient [73]. However, there are also diverse findings, where a case reported a new homozygous splicing mutation in the MRPS22 gene c.339+5G>A in a patient with mild dysmorphism, hypotonia, and developmental delay. However, this patient did not develop hypertrophic cardiomyopathy and tubal disease [129]. Pathogenic mutations in *MRPL3* were responsible for the clinical phenotype of combined oxidative phosphorylation deficiency-9 (COXPD9), in which severe hypertrophic cardiomyopathy is a central presentation [4]. *mrpl16* has been shown to be significantly associated with septic cardiomyopathy [17]. The genetic variation in *MRPL37* was linked to an increased risk of venous thromboembolism recurrence [37]. Meng et al. designed the cardiac hypertrophy cells by culturing the cardiac cells with 25 mmol/L D-glucose for 48 h. With the help of quantitative real-time PCR, it was confirmed that the expressions of mrpl50, mrps2 and mrps21 were upregulated, and mrpl34 was downregulated [33]. According to Bayesian multi-trait approaches and the Bayesian network analysis, *MRPL38* was found to have significant effects on the cardiac traits such as left ventricular volume index, parasternal long axis interventricular septum thickness and mean left ventricular wall thickness [130].

### 5.4. MRPs Associated with Age and Other Related Mitochondrial Diseases

An abnormal MRPs expression was closely related with age. The specific downregulation of mrpl2 in the retina was identified in elderly APP/PS1 mice aged 8 months [131]. The expression of mrpl4 decreased after a short period of fasting treatment and the methylation level of mrpl4 increased with age through the epigenetic map in mice [105]. A single nucleotide polymorphism rs3209 in *MRPL10* associated with early age-related macular degeneration (AMD) was also reported in Chinese Americans [9]. However, other researchers have taken a different view, claiming they found no link between MRPs and longevity [132].

The association between abnormal MRPs expression and other diseases have been gradually confirmed. The DNA methylation of *MRPS18-B* reduced survival in patients with tuberculosis and might be a determinant of long-term outcomes [69]. *MRPL44* is associated with a variety of asthma and allergy-related traits [48]. *MRPL3* is one of the recently discovered hub genes associated with acute mountain sickness, which may be used as a biomarker and therapeutic target for an accurate diagnosis and treatment in the future [7]. MRPL36 is significantly associated with the meow syndrome by analyzing the protein interaction network [36]. Moreover, MRPs can have a positive effect on the treatment of diseases. *CRIF1 (MRPL64)* can regulate the oxidative stress of irradiated bone marrow mesenchymal stem cells through phosphorylation of NRF2^Ser40^, which is helpful for the treatment of hemocytopenia, as well as multiple organ failures caused by hematopoietic dysfunction in the acute radiation syndrome [56]. CRIF1 (MRPL64) interacting with CDK2 can enhance the radio-sensitivity of tumor cells [133].

## 6. Conclusions and Perspectives

The development of a high-precision analysis technology of cryo-electron microscopy enables us to identify the structure of mitochondrial ribosomal proteins with a scale of 0.1 nm. Each of the 80 MRPs is essential for the mitochondrial ribosome composition, which plays an irreplaceable role in the assembly and translation of mitochondrial DNA. At present, studies of the relationship between MRPs and cell apoptosis are few. Although the apoptotic mechanisms of MRPS29, MRPL41 and MRPL65 have not been fully elucidated, they at least provide us with useful information to deeply study the apoptotic mechanisms of MRPs. We can further explore the changes of MRPs function or pathway-activating role in the process of inducing apoptosis, based on the analysis of the MRPs structure. Additionally, specific mechanisms can be clarified in the future.

The abnormal expression of MRPs and their encoding genes is closely associated with a variety of cancer and mitochondrial related diseases. Multiple MRPs are important predictors of disease diagnosis. However, the specific mechanisms of inducing the development of diseases is little known.

In the future, on the one hand, it is very important to strengthen the research on the relationship between the abnormal expression of MRPs, lack of their encoding genes, and diseases. On the other hand, some MRPs such as MRPS22, MRPL44 and MRPL28 that have been clarified as key factors in the development of cancer, which can be as biological targets to deeply study their specific pathways of influence, in order to lay a theoretical foundation for a targeted diagnosis and therapy of cancer in our research.

## Figures and Tables

**Table 1:** Summary of the relationship between abnormal expression of mitochondrial ribosomal proteins and their encoding genes with diseases in recent 5 years.

Old Name	New Name	Cancer	Other Diseases
Mitochondrial ribosomal large subunit (mt-LSU) genes and proteins.			
MRPL1	uL1m	Lung cancer [2]	
MRPL3	uL3m		Neurodegeneration and memory impairment [3], Hypertrophic cardiomyopathy [4,5], Prognosis [6], Acute mountain disease [7]
MRPL9	bL9m	Breast cancer [8]	
MRPL10	uL10m		Early age-related macular degeneration [9]
MRPL11	uL11m		Mitochondrial encephalopathy [10] ↓
MRPL12/L7	bL12m	Breast cancer [11] ↑, Colorectal cancer [12]	
MRPL13	uL13m	Liver cancer [13] ↓, Breast cancer [13,14,15] ↑	
MRPL15	uL15m	Breast cancer [16] ↑	
MRPL16	uL16m		Septic cardiomyopathy [17] ↑
MRPL17	bL17m	Lung cancer [18] ↑	
MRPL19	bL19m	Endometrial cancers [19,20], Diffuse non-Hodgkin lymphoma [21], Melanoma [22]	
MRPL21	bL21m	Acute myeloid leukemia [23] ↑	
MRPL23	uL23m	Oral squamous cell carcinoma [24] ↓, Glioblastoma multiforme [25]	
MRPL24	uL24m		Cerebellar atrophy, intellectual disability [26] ↓
MRPL28	bL28m	Gastric cancer [27]	
MRPL33	bL33m	Breast cancer [28] ↑, Lung cancer, colon cancer [29] ↑, Gastric cancer [30], Acute myeloid leukemia and neuroblastoma [31] ↑, Human papillomavirus associated oropharyngeal squamous cell carcinoma [32] ↑	
MRPL34	bL34m		Cardiomyocyte hypertrophy [33] ↓
MRPL35	bL35m	Glioblastoma multiforme [34] ↑, Colorectal cancer [35] ↑	
MRPL36	bL36m		Cri-du-chat syndrome [36]
MRPL37	mL37		Venous thromboembolism [37]
MRPL38	mL38	Liver cancer [38]	
MRPL39	mL39	Gastric cancer [39] ↓	
MRPL40	mL40		Schizophrenia [40,41,42] ↓
MRPL42	mL42	Glioma [43] ↑	
MRPL43	mL43	Gastric cancer [44] ↑	
MRPL44	mL44		Mitochondrial encephalopathy [10] ↓, Cardiomyopathy [45,46]. Hemiplegia migraine, pigmentary retinopathy, renal insufficiency, Leigh-like lesions on brain MRI [47], Asthma and allergy-related traits [48]
MRPL46	mL46	Ovarian cancer [49]	
MRPL47	mL47	Acute lymphoblastic leukemia [50]	
MRPL50	mL50		Cardiomyocyte hypertrophy [33] ↑
MRPL51	mL51	Lung cancer [51] ↑	
MRPL52	mL52	Colorectal cancer [52] ↓	
MRPL54	mL54	Breast cancer [53]	
CRIF1	mL64	Hepatocellular carcinoma [54] ↓, T-cell leukemia [55]	Acute radiation syndrome [56], Endothelial inflammation [57,58] ↓, Autoimmune arthritis [59] ↑
MRPS18-A	mL66	Liver cancer [60] ↑, Cholangiocarcinoma [61] ↑	
Mitochondrial ribosomal small subunit (mt-SSU) genes and proteins			
MRPS2	uS2m	Glioblastoma multiforme [25]	Cardiomyocyte hypertrophy [33] ↑
MRPS5	uS5m		Noise-induced hearing loss and anxiety related behavior changes [62] ↑
MRPS7	uS7m	Osteosarcoma [63] ↑	Primary hypogonadism, primary adrenal failure [64] ↓
MRPS11	uS11m	Uveal melanoma [65] ↑	
MRPS12	uS12m	Glioblastoma multiforme [25]	
MRPS14	uS14m		Perinatal hypertrophic cardiomyopathy [66] ↑
MRPS18-B	mS40	Prostate cancer [67] ↑, Colorectal carcinoma [68]	Tuberculosis [69]
MRPS18-C	bS18m	Breast cancer [70] ↑	Epileptic encephalopathy [71]
MRPS21	bS21m		Cardiomyocyte hypertrophy [33] ↑
MRPS22	mS22		Epicanthus inversus syndrome [72], Hypertrophic cardiomyopathy and fallopian tube lesions [73] ↓, Primary ovarian insufficiency [74,75,76]
MRPS23	mS23	Hepatocellular carcinoma [77] ↑	
MRPS34	mS34	Glioblastoma multiforme [25]	Cardiomyocyte hypertrophy [33] ↓
MRPS37	mS37	Acute lymphoblastic leukemia [23] ↑	
MRPS39	mS39		Leigh syndrome [78]

**Legend**: Prefix “u”: Genes and proteins are present in all kingdoms of life (for universal); prefix “u”: Genes and proteins are bacterial in origin and do not have an eukaryotic (or archaeal) homolog; prefix “m”: Genes and proteins are mitochondrion-specific. “↑” Upregulation in that disease; “↓” downregulation in that disease. This table only lists the MRPs (mitochondrial ribosomal proteins) that appear in this article.
